# Nuclear Integrity but Not Topology of Mouse Sperm Chromosome is Affected by Oxidative DNA Damage

**DOI:** 10.3390/genes9100501

**Published:** 2018-10-17

**Authors:** Alexandre Champroux, Christelle Damon-Soubeyrand, Chantal Goubely, Stephanie Bravard, Joelle Henry-Berger, Rachel Guiton, Fabrice Saez, Joel Drevet, Ayhan Kocer

**Affiliations:** GReD “Genetics, Reproduction & Development” Laboratory, UMR CNRS 6293, INSERM U1103, Université Clermont Auvergne, 28 Place Henri Dunant, 63000 Clermont-Ferrand, France; alexandre.champroux@uca.fr (A.C.); christelle.soubeyrand-damon@uca.fr (C.D.-S.); Chantal.goubely@uca.fr (C.G.); Stephanie.bravard@uca.fr (S.B.); joelle.henry@uca.fr (J.H.-B.); rachel.guiton@uca.fr (R.G.); fabrice.saez@uca.fr (F.S.)

**Keywords:** mouse sperm chromatin, chromosome organization, nuclear-3D-parameters

## Abstract

Recent studies have revealed a well-defined higher order of chromosome architecture, named chromosome territories, in the human sperm nuclei. The purpose of this work was, first, to investigate the topology of a selected number of chromosomes in murine sperm; second, to evaluate whether sperm DNA damage has any consequence on chromosome architecture. Using fluorescence in situ hybridization, confocal microscopy, and 3D-reconstruction approaches we demonstrate that chromosome positioning in the mouse sperm nucleus is not random. Some chromosomes tend to occupy preferentially discrete positions, while others, such as chromosome 2 in the mouse sperm nucleus are less defined. Using a mouse transgenic model (*Gpx5*^−/−^) of sperm nuclear oxidation, we show that oxidative DNA damage does not disrupt chromosome organization. However, when looking at specific nuclear 3D-parameters, we observed that they were significantly affected in the transgenic sperm, compared to the wild-type. Mild reductive DNA challenge confirmed the fragility of the organization of the oxidized sperm nucleus, which may have unforeseen consequences during post-fertilization events. These data suggest that in addition to the sperm DNA fragmentation, which is already known to modify sperm nucleus organization, the more frequent and, to date, the less highly-regarded phenomenon of sperm DNA oxidation also affects sperm chromatin packaging.

## 1. Introduction

The mammalian spermatozoon is a highly-differentiated cell produced by the testis during a long and complex process called spermatogenesis. Following successive steps that lead to the multiplication and the production of haploid germ cells through the meiotic program, spermatids undergo a long phase of cyto-differentiation (the so-called spermiogenesis phase) to form highly polarized spermatozoa. Unique characteristics of these cells are featured by the quasi-complete loss of the cytoplasmic content, appearance of the flagella apparatus and drastic size reduction of the nuclear compartment. These major cytological changes give rise to the tiniest mammalian cell type that has the ability to move in order to fulfil its function of delivering to its target, the oocyte, the compacted and, consequently, protected paternal genomic moiety. Up to the spermatid stage the germ cell chromatin presents a somatic organization consisting of short (147 bp) DNA segments wrapped around a histone octamer to form a nucleosome [1]. During spermiogenesis, most (but not all) canonical histone core proteins (H3, H4, H2A, and H2B) are replaced by testis-specific histone variants such as TH2B, H3t, H2AL2 & 5 [2,3,4,5]. It is assumed that the inclusion of such variants allows a more dynamic chromatin structure that permits the upcoming changes. Subsequently, histones, both canonical and testicular variants, are largely replaced by small basic proteins called transition nuclear proteins (Tnps), and find themselves replaced by even smaller and more basic proteins called protamines [6,7]. Protamines and DNA organize themselves into a ring-shaped structure called a toroid, containing up to 100 kb of DNA that ultimately piles up along the chromosomes, greatly increasing the level of the DNA compaction [8,9,10,11]. This sequence of events allows a strong nuclear and cell size reduction, when compared to any somatic cell [12]. Together with the fact that these modifications enable optimization of cell mobility, they also contribute to passive protection of the paternal sperm genome in anticipation of its long post-testicular journey to the site of fertilization [13].

Another unique feature of this reshaping of the mammalian sperm, chromatin, is that the supra-organization of the chromosomal chromatin is also tightly ordered and conserved from one sperm cell to another. This has led to the observation that chromosomes are not randomly distributed in the sperm nucleus and that they occupy domains, called chromosome territories (CTs) [14,15,16]. A limited number of species have been investigated, to date, and for those analyzed (mainly human) not all chromosomes were mapped in the sperm nucleus, with the exception of the porcine sperm [14]. The localization of specific chromosomal regions such as telomeres and centromeres were also investigated in the human sperm nucleus [17,18]. As is the case in somatic cells, sperm cell chromosomes are attached to a nuclear protein scaffold, called the sperm nuclear matrix, which consolidates the structure [19,20,21]. Here too, the manner in which chromosomes are attached to the sperm nuclear matrix is unique to that cell lineage and is dissimilar to the somatic situation [19,22]. Two non-exclusive theories have been proposed to explain the positioning of chromosomes in the nucleus of a somatic cell. The first is “gene density” with the assumption that gene-poor chromosomes orient themselves toward the nuclear periphery while gene-rich chromosomes are located toward the nuclear interior [23,24]. The second theory, and in our opinion the more pertinent, takes chromosome size into account since, at least in the human sperm, it appears that small chromosomes are located in the center of the nucleus while larger chromosomes are located at the periphery [16,25,26]. Whether the human sperm nuclear organization reflects that of other mammals is a matter of debate.

For many years it was reported that mature spermatozoa do contain residual histones and that the quantity of the so-called persisting histones was species-specific. Indeed, it was estimated that about 1–2% of mouse, hamster, and bull sperm DNA was still associated with histones [27,28,29] and that this value increased to 15% in human sperm [30,31,32,33,34]. First, attributed to an incomplete, therefore deficient, spermiogenesis program, it was recently reported that persisting histones in the sperm nucleus were not random, but were deliberately excluded from the histone-to-protamine exchange. Although, there is a controversy regarding the extent and quality of nucleosome retention in mammalian spermatozoa it is clear that histones are found in large domains punctuating the protamine-toroidal stacks along the chromosomes and, in addition, nucleosomes persist at each small string of DNA, connecting the adjacent toroids [20]. The consensual explanation for this situation is that these particular paternal regions that maintain a somatic-like organization will be more prone to reactivation early after fertilization at the onset of the developmental program. In support of this hypothesis were the observations that the genes important for the early developmental program were found located in such histone-containing regions [30,31,32], and that the origins of the paternal DNA replication necessary, prior to the first division of segmentation, were located in the short histone-containing DNA segments, connecting the toroids and is attached to the nuclear matrix [19,35,36,37,38]. It is thought that this ordered-organization of the paternal chromosomes in the sperm nucleus is essential after fertilization, during the sequential decondensation phase of the male nucleus into the male pronucleus [16,39].

In recent years, we have shown in a mouse model that these histone-rich regions, particularly those that are attached to the nuclear matrix were mainly localized at the sperm nuclear periphery and at the base of the sperm nucleus towards the so-called annulus domain [35,40]. In agreement with the lower level of condensation and the peripheral easy access of these histone-associated DNA domains we also demonstrated that these regions were particularly susceptible to DNA damage and in particular to oxidative DNA damage [35]. We also reported that smaller chromosomes were highly susceptible to DNA oxidation [41] in the mouse sperm nucleus. We demonstrated that this was not related to their content of persisting histones, but rather to the more peripheral and basal position of small chromosomes [36]. These observations led to the conclusion that in contrast to human sperm chromosomal organization, which as mentioned above, showed small chromosomes, located more in the central axis of the sperm nucleus, the situation was different in the mouse. This prompted a more precise analysis of the architecture of the mouse sperm nucleus. In the present study, we used three-dimensional fluorescence in situ hybridization (3D-FISH), confocal microscopy, and computational analysis of 3D structures to analyze the topology of at least twelve mouse sperm chromosomes. This has allowed us to propose the largest map of chromosome territories in murine sperm, to date. Our access to *Gpx5*^−/−^ transgenic mice, in addition to wild-type controls, allowed us to conduct an analysis of chromatin organization in what now appears to be a frequent type of sperm nuclear damage, i.e., nuclear oxidation [42]. This mouse model was very pertinent to address this question because we reported earlier that *Gpx5*^−/−^ males present mild oxidative sperm DNA damage that does not translate to an increase in either sperm DNA fragmentation or nuclear decondensation. This transgenic mouse model was particularly interesting, therefore, as it dissociates the effect of severe sperm DNA damage from the low-grade DNA oxidation situation commonly seen in infertile patients. Indeed, we recently demonstrated that males in two-thirds of couples entering an infertility program, showed mild to severe sperm DNA oxidation. Our aims were then to investigate whether chromosomal 3D parameters including volume and surface area would be affected by DNA oxidation.

## 2. Results

### 2.1. Localization of Chromosome Territories in Murine Spermatozoa

**Figure 1:**
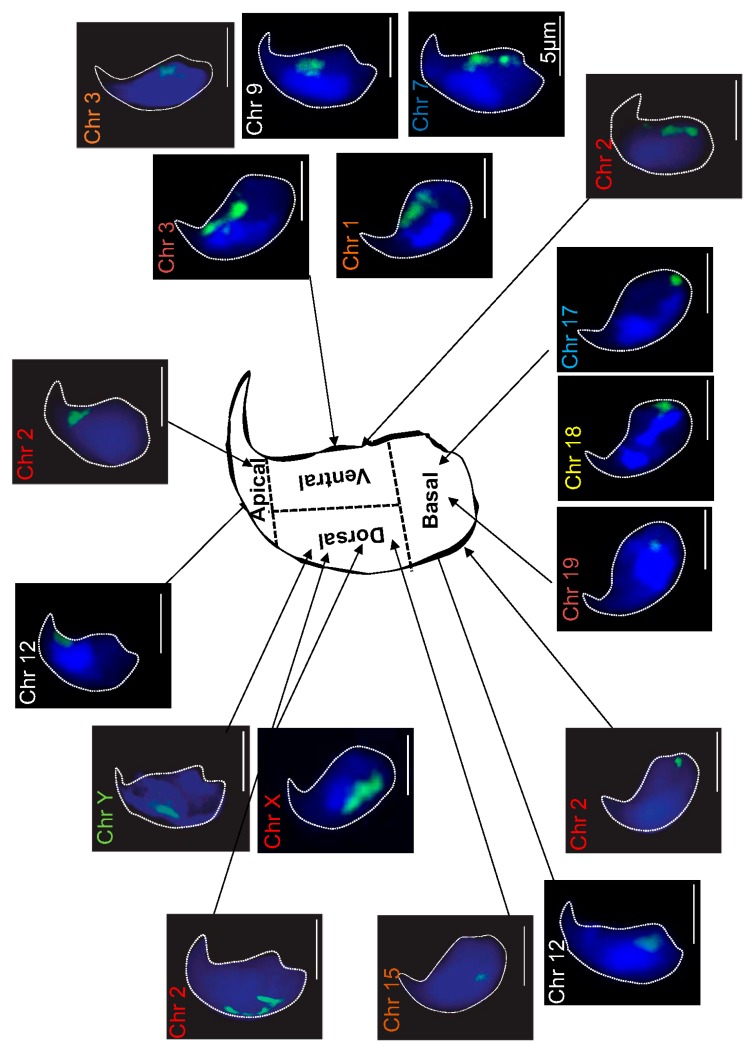
Chromosome mapping in WT mouse sperm nucleus. Schematic representation of a wild-type (WT) mouse sperm nucleus, arbitrarily divided into four regions (apical, dorsal, ventral, and basal). The position of each selected chromosome was detected by fluorescence in situ hybridization (FISH). Green (FITC) staining represents the chromosome position (n = 350 spermatozoa). Nuclei were stained blue with DAPI. Nuclei were captured in Z-stacks by using confocal microscopy and subjected to deconvolution (Huygens software, Hilversum, The Netherlands). Scale bar represents 5 μm (white line). Chr: Chromosome.

Previously, we hypothesized that the localization of chromosomes, in the mouse sperm nucleus, could explain their different susceptibility to oxidative damage, as revealed after immunoprecipitation of the oxidized DNA regions, followed by high throughput sequencing approaches [41]. This statement was supported by the fact that we were able to co-localize the smallest murine chromosome (chromosome 19), with a focal point of oxidative DNA damage, in the *Gpx5^−/−^* sperm nucleus [41]. To lend support to this statement, we looked at the nuclear distribution of a total of twelve chromosomes (both long and short chromosomes) using the *FISH* assay, in a whole chromosome-painting approach, in both WT and *Gpx5*^−/−^ sperm nuclei. Figure 1 shows representative confocal microscopy photographs going through the middle of the sperm head for each chromosome investigated. To facilitate this analysis, we arbitrarily divided the mouse sperm head into four distinct areas, as schematized in Figure 1. For each selected chromosome, a minimum of three hundred and fifty sperm cells were analyzed and preferential chromosome positions were determined. It is clear that the small chromosomes, including chromosomes 17, 18, and 19, localized to the basal part of the sperm nucleus, whereas a long chromosome, such as chromosome 1, localized preferentially to the ventral area (see Figure S1, supplemental data). Chromosome 15 and the X and Y sex chromosomes also clearly localized to the dorsal area (Figure 1). Assignation to a preferential domain was easy for these chromosomes because a clear preference was found for these particular locations (see Table 1). In contrast, assignation to a preferential area was more difficult for some chromosomes. For example, two chromosomes (3 and 12) were statistically equally-assigned to two sperm head areas, namely, basal and ventral for chromosome 3 and basal and apical for chromosome 12 (Table 1). Chromosome 2 was peculiar as it was equally localized among the four distinct areas (Table 1). When the same analysis was carried out using *Gpx5*^−/−^ oxidized sperm, it was clear that no difference was recorded (see Table 1).

Taking advantage of the 3D-reconstructed images we examined two topological parameters (volume and surface area), for each chromosome in the WT genetic background. As shown in supplemental Table S1 and supplemental Figure S1, it is clear that there is a linear relationship between the size of a given chromosome and the volume/surface it occupies in the mouse sperm nucleus. Only chromosome 2 behaved in a peculiar manner, since the linear relationship was validated in only 25% of the analyzed sperm—those in which chromosome 2 localized to the basal area (B in supplemental Table S1 and supplemental Figure S1). Strikingly, when chromosome 2 localized to different areas of the sperm nucleus the linear relationships (volume vs. size and surface vs. size) were lost (supplemental Figure S1). This was particularly true when chromosome 2 was located in the ventral (V) and apical (A) areas and to a lesser extent in the dorsal (D) area. Interestingly, contrasting effects were recorded in these two situations, revealing that when chromosome 2 localized to the ventral and apical areas of the sperm nucleus, its footprint (volume/surface) in the sperm nucleus differed from that when localized to the basal area.

### 2.2. Centromeres, Telomeres, and Histone-Rich Domains Clustered in the Mouse Sperm Nucleus

**Figure 2:**
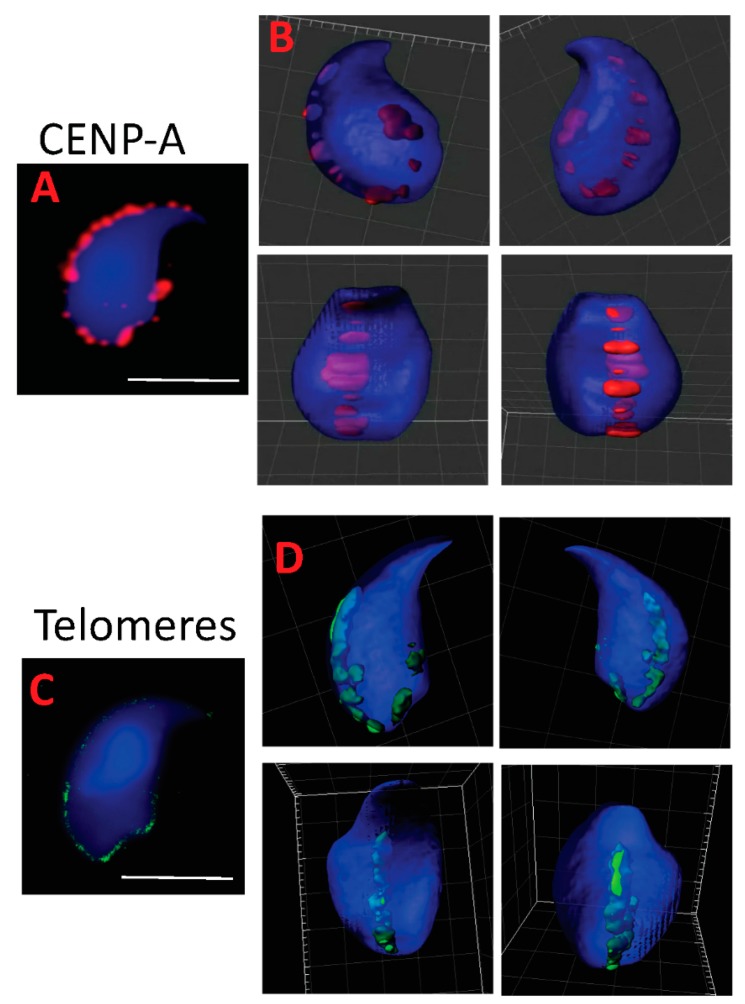
Representative image of telomere and centromere positions in WT mouse sperm nucleus. The centromere-specific histone H3 variant (CENP-A, red (**A**,**B**)) and telomeric probes ((**C**,**D**), red) were used in immunofluorescence or FISH approaches, respectively. Nuclei were stained blue with DAPI. Nuclei were captured in Z-stack, using confocal microscopy, and subjected to deconvolution (Huygens software, Netherlands). The 3D models were obtained with Imaris software (Bitplane, Switzerland). The set of views per staining represented is a representative nucleus from a pool of 30 spermatozoa. Scale bar in confocal images represents 5 μm (white line).

**Figure 3:**
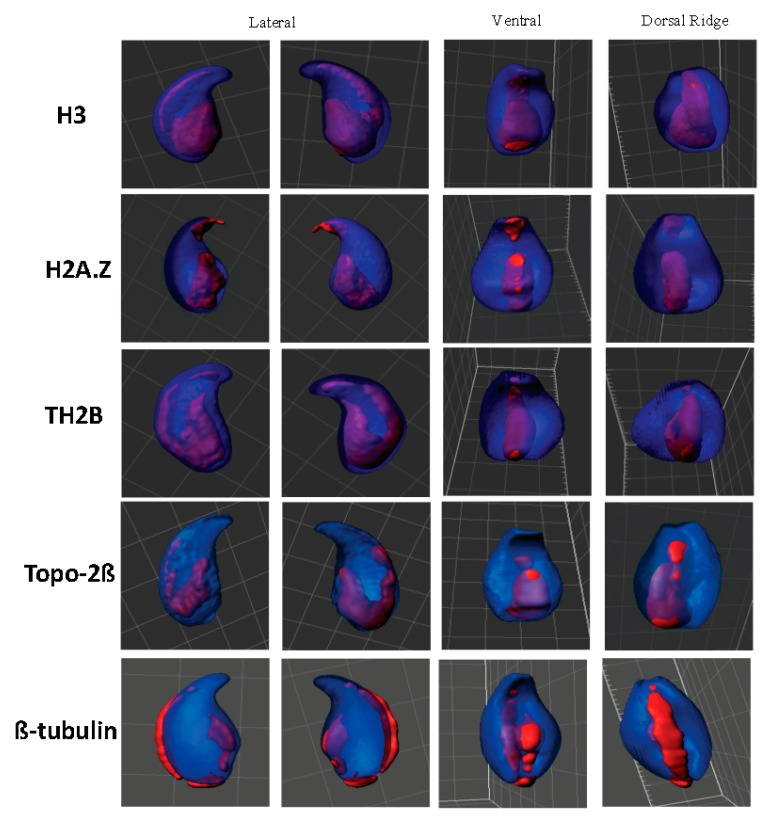
Representative image of chromatin components in WT mouse sperm nucleus. Representative confocal and different views are shown for each component of sperm chromatin in mouse sperm nucleus: Histone H3, histone variant H2A.Z, testis-specific histone variant TH2B, nuclear matrix protein Topoisomerase-II, and ß-tubulin in WT mouse sperm nucleus. Nuclei are captured in Z-stacks using confocal microscopy and subjected to deconvolution (Huygens software, Netherlands). The 3D models were obtained with Imaris software (Bitplane, Switzerland). The set of views per component is a representative nucleus of thirty spermatozoa.

Using immunocytochemistry and *FISH*, we further investigated the localization of particular chromosomal subdomains, namely centromeres and telomeres. To do so, we used a pan-centromere specific H3 variant (CENP-A) antibody to detect this ubiquitous centromeric protein (Figure 2A). 3D reconstruction using Imaris software showed that centromeres aligned and clustered along the dorsal and basal ridges of the sperm head (Figure 2B). A similar localization was observed by *FISH* when looking at telomeres (Figure 2C,D) suggesting that in the mouse sperm nucleus, centromeres and telomeres co-localize. No difference in the localization of centromeres and telomeres was recorded when *Gpx5^−/−^* sperm nuclei were examined (data not shown). We used three specific histone antibodies (1 canonical and 2 testis-specific variants, respectively, H3, TH2B, and H2A.Z) to corroborate and complete earlier reported partial observations [35] regarding the localization of persisting histones in the mouse sperm nucleus, in immunofluorescence confocal microscopy approaches, associated with 3D Imaris reconstruction. We confirm the basal and dorsal peripheral localization of these persisting histones and their consistently overlapping localization (Figure 3). The 3D Imaris reconstruction, shown in parallel (right panels) in the same Figure, clearly reveals the basal and dorsal ridge localization of these histone-rich domains in what could be designated a “punk-head” distribution. Topoisomerase 2ß, a sperm nuclear matrix protein (Figure 3), as well as the classical cytoskeleton protein ß-tubulin (Figure 3), also fall into these dorsal peripheral and basal ridge domains as was partly shown in the earlier study [30].

### 2.3. Oxidative DNA Damage Does Affect 3D-Parameters of the Mouse Sperm Nucleus

**Figure 4:**
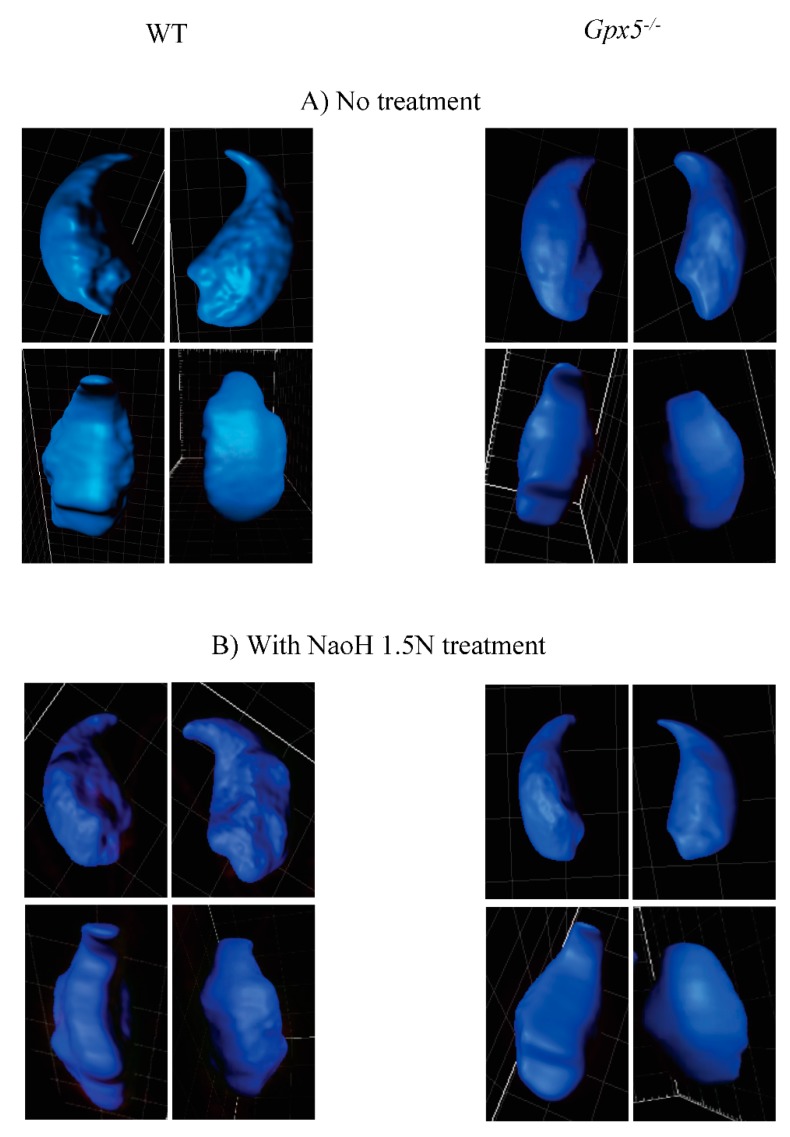

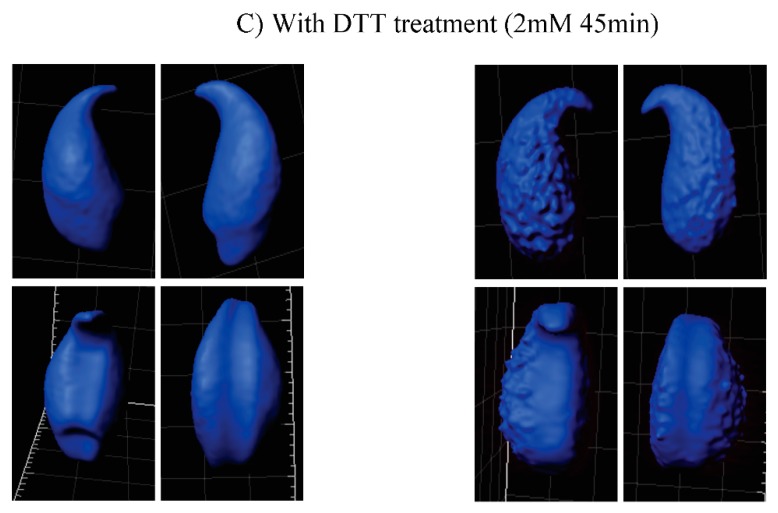
NaOH-mediated or DTT-mediated mild denaturation provokes distinct effects on the WT and the *Gpx5^−/−^* nuclei.

Taking advantage of the confocal images and the power of the Imaris software analysis, we looked in more detail at sperm nuclear 3D-parameters, including volume and surface area, comparing WT and *Gpx5^−/−^* spermatozoa. An average value for each parameter (volume and surface area) was obtained from each sample and each condition tested (untreated, NaOH- or DTT-treated) by looking at a pool of thirty spermatozoa. The data are presented in Table 2. Untreated WT spermatozoa showed a mean nuclear volume of 66 µm^3^ and a mean nuclear surface area of 93.9 µm^2^. These parameters were significantly different in *Gpx5^−/−^* spermatozoa, which had a mean nuclear volume of 54.8 µm^3^ (*p* < 0.001) and a mean surface area of 80.2 µm^2^ (*p* < 0.001), revealing a greater state of nuclear condensation. Examination of the detailed shape of the 3D-reconstructed sperm nuclei revealed repeated differences between the WT and *Gpx5^−/−^* animals. As shown in Figure 4, with representative photographs of 3D-reconstructed nuclei, *Gpx5^−/−^* sperm nuclei present a smoother surface when compared to the more irregular aspect of the WT sperm nuclei. The use of different mild denaturing treatments, namely DTT (2 mM) or NaOH (1.5 N), revealed distinct reactions when WT sperm were compared to *Gpx5^−/−^* sperm and confirmed the specific effect of oxidation on the sperm nucleus. As presented in Table 2, when NaOH was used to produce a mild denaturation of the sperm chromatin (by classical breakage effects on the hydrogen bonds linking DNA base pairs), we recorded/*observed* a significant increase in sperm nuclear volume and surface area, in both genetic backgrounds (WT and *Gpx5^−/−^*). However, *Gpx5^−/−^* sperm nuclei remained more condensed than WT following treatment with alkali. In contrast, when DTT (a non-ionic detergent that specifically reduces disulfide bonds to free thiols) was used, we observed a marked effect on both sperm nuclear volume and surface area, in the *Gpx5^−/−^* mice, as compared with WT controls (Table 2). This is in agreement with the idea that although *Gpx5^−/−^* sperm nuclei appear more condensed, they also appear to be significantly less robust when exposed to a mild, reducing environment. These differences in the nuclear reactivity of oxidized or non-oxidized sperm nuclei, when exposed to mild denaturing conditions, can be visualized, as shown in Figure 4. In panel C (Figure 4C), when no denaturing treatment was performed, the *Gpx5^−/−^* sperm nuclei presented the smooth aspect, as noted above. When NaOH was used as a mild denaturing treatment, there was no significant change regarding the smooth shape of the sperm nuclei in either genetic background (Figure 4B). However, when mild denaturation was carried out with DTT, it was obvious that the *Gpx5^−/−^* sperm nuclei then presented a dense granular aspect (Figure 4C) that was not observed in the WT.

## 3. Discussion

In recent years, it has become apparent that mammalian sperm nucleus organization has implications for fertilization and early embryogenesis [14,15,43,44,45,46]. It was shown, mainly in human spermatozoa, that most chromosomes occupy discrete and well-defined territories in a polar/radial distribution that could be partly related to their size [44,45,46], the shape/volume of the mature sperm cell and the kinetics of the oocyte-driven decondensation program of the paternal nucleus post-fertilization [47,48]. How this highly-ordered organization of the sperm chromatin is achieved, controlled, and maintained in each sperm cell, throughout spermiogenesis and beyond, is still largely unknown. Whether the human sperm chromatin organization applies to murine sperm and how susceptible this organization is to mild nuclear and DNA damage, as represented by the common situation of sperm DNA oxidative damage, are questions we addressed in this study.

Using FISH experiments, we determined the position of a total of twelve chromosomes in the mouse sperm nucleus. Both short and long autosomes and the two sex chromosomes were analyzed. As reported for the human sperm nucleus, and suggested for other species (including mouse, bovine, pig, and rat), using a smaller subset of chromosome probes when compared to the present work [14,15,16,17,45,49,50,51,52,53,54,55,56], chromosome positions in the mouse sperm nucleus were not random. This situation seems to be confined to mammals since a tandem head-to-tail organization of sperm chromosomes, in a defined order, was observed in monotremes and marsupials [57,58] while no particular organization was detected in non-mammals, including chicken and planarian spermatozoa [59,60].

Due to this peculiar, asymmetric hook-shape morphology of the mouse sperm head it was difficult to use a polar/radial axis to map the mouse sperm head, as has been performed in other species [15]. We arbitrarily separated the mouse sperm head into four compartments (apical/basal/dorsal/ventral), while still permitting comparative analyses with other species. In the mouse, smaller chromosomes were found to occupy a basal localization, whereas longer chromosomes were preferentially found in the ventral area with the sex chromosomes located in the dorsal area of the sperm nucleus. This appears to be distinct from the human situation since it was shown that small autosomes as well as sex chromosomes occupy a rather central position in human sperm [16]. Some of the CTs appear to be small while others are larger. Our assumption is that it is both related to the respective size of the chromosomes (since we did observe that there is a positive correlation between the size and the volume of the chromosome, as shown in supplementary Table S1). However, it could also be partly related to the number of times by which the chromosomes—which are folded to fit into the tiny nuclear volume—are longer. Although a preferential position could be assigned for most of the chromosomes examined, this did not hold for all chromosomes. Four chromosomes (chr 3, 7, 9, and 12) were equally assigned to two distinct areas, while one chromosome (chr 2) was very plastic and was found evenly distributed among the four arbitrarily-defined nuclear areas. For those chromosomes that were equally distributed between the two distinct nuclear domains, one explanation could arise from the fact that statistically one out of two spermatozoa examined was either a Y-spermatozoon or an X-spermatozoon. The size difference between the sex chromosomes (both localized in the dorsal area) could explain the alternate positions of these autosomes. This hypothesis is strengthened by the observation that overall Y-sperm and X-sperm show a similar nuclear volume (not shown here) suggesting that the necessary adjustment to accommodate the X or Y chromosome size-difference does not rely on nuclear volume variation. Furthermore, when looking at individual chromosome 3D-parameters (i.e., volume and surface area) we observed that chromosomes 3 and 12 (two chromosomes that show equal occupancy of two distinct locations, basal or ventral for chromosome 3 and basal or apical for chromosome 12) have the same footprint, irrespective of their location. This suggests that the nuclear space adjustment necessary to accommodate the X or the Y chromosome does not rely on different folding of individual chromosomes, but rather on different chromosome positions. These hypotheses would require verification using a triad-detection system with probes targeting a chosen autosome, together with probes targeting sex chromosomes. Chromosome 2 is rather intriguing as it distributes equally in any of the four arbitrarily defined nuclear areas. This observation is not unique to murine sperm, since human sperm chromosome 13 showed identical behavior [17]. Although a rather long autosome, it seems that chromosome 2 is considered as an adjustment variable in the mouse sperm nucleus. In addition, we and others have data suggesting that mouse chromosome 2 is a rather accessible chromosome in the mouse sperm nucleus, since it was observed on several occasions that, when purifying murine sperm DNA for high throughput sequencing strategies, one systematically obtained a large excess of chromosome 2 sequences in comparison to other chromosomes [41,61,62]. This suggests a peripheral localization of this chromosome in the mouse sperm nucleus as it does not appear to be less-condensed than other autosomes [41].

**Figure 5:**
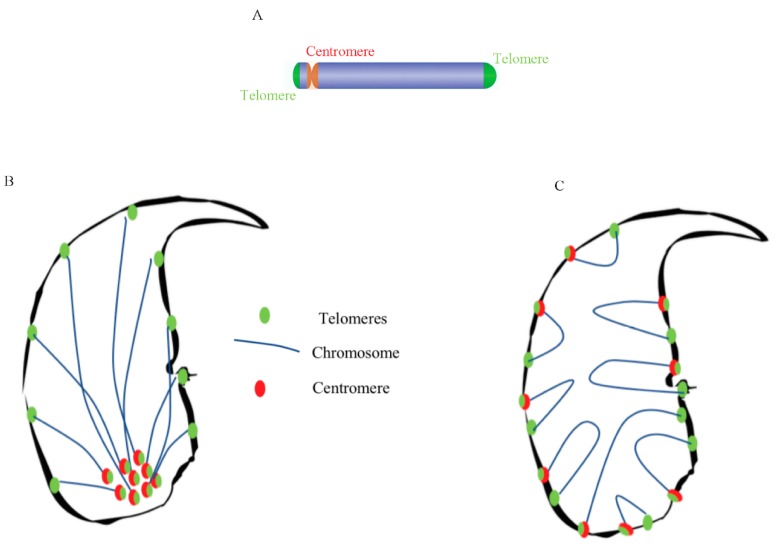
Schematic representation of the proposed models of telomere and centromere organization within murine sperm nuclei. Panel (**A**) presents a schematic representation of the murine acrocentric chromosome with two telomere regions (green) at either end of the chromosome and one centromere (red). Panel (**B**) presents a schematic representation of the murine chromosome model in which the centromeres (red) gather in a chromocenter, with the chromosome (light blue) stretching out toward the telomere (green) localized at the peripheral region. In panel (**C**), we present a refined version of the model, based on our observations, which depicts a more segmented organization, with localization of telomeres (green) and centromere (red), throughout the murine nucleus.

Telomeres have recently been assigned a chromosome stabilizing function that is important for reproduction [63] and it is proposed that telomeres are the first chromosomal regions to respond to oocyte decondensing factors that lead to the formation of the male pronucleus [46]. As suggested earlier in mouse sperm nucleus [49,56] and recently confirmed for the human sperm nucleus [17,52,56,64], we showed here that telomeres in murine sperm are also organized in clusters located at the periphery of the sperm nucleus in an edge-like/ridge-like manner, starting from the base of the nucleus and extending along the dorsal side, in close proximity to the peripheral nuclear matrix. With regard to centromeres, another characteristic domain of chromosomes rich in repeated sequences we found that in the mouse sperm nucleus, they were also located in clusters, at the periphery, with the same edge-like/ridge-like organization. In a previous study, it was shown via *FISH* that the distribution of centromeres in testicular sperm (not fully mature) are clustered at the surface of the heterochromatic chromocenter (schematic representation in Figure 5B). This differed from our study [49] in which fully mature post-testicular (i.e., epididymal) sperm were evaluated. An organization similar to the one we report here was recently described in human sperm nuclei, in which the centromeres were distributed as single clusters [64]. The present localization of centromeres in murine sperm, determined by using the histone H3 variant CENP-A, is in agreement with previous data reporting that histones in mature murine sperm are preferentially located in the basal and dorsal peripheral areas of the nucleus [35]. In view of these results we propose a new model for telomere and centromere organization in murine sperm nuclei (Figure 5C). It would appear that in the mouse, both telomeres and centromeres are closely located at these dorsal peripheral and basal nuclear domains that were shown elsewhere (as well as here) to be domains rich in nuclear matrix attachment components [30] and rich in histone [65,66]. It is interesting to note that the paternal DNA associated with these nuclear regions was shown to be important both for male pronucleus formation and for the first round of DNA replication [19,37] which are early events of embryo development. As it has been well described in a recent review [67], the organization of the sperm nucleus seems to be an important factor for male fertility and embryo development that will require further analysis.

Concerning the susceptibility of the sperm chromatin organization to oxidative alterations, gross examination of the nuclear topology of the chromosomes (studied in this work) shows that they are unaffected by the mild oxidative environment present in the *Gpx5^−/−^* transgenic mouse strain. This is supported by the fact that we did not record significant differences in the distribution of the chromosomes, in the four arbitrarily defined regions, when comparing WT and transgenic sperm (Table 1). However, a recent study did show that high levels of DNA damage in human sperm (such as significant DNA fragmentation) could disrupt the position of the centromeres [68]. This suggested that chromosome 3D organization may be impacted depending on the level of sperm DNA damage. 

When looking at nuclear 3D-parameters, such as nuclear volume and surface area we confirmed, as expected, the susceptibility of the nucleus to oxidative alterations. This is evidenced by the observations that both nuclear volume and nuclear surface area are significantly diminished in the *Gpx5^−/−^* spermatozoa, when compared with WT sperm. This is in line with the idea that when the epididymis-secreted GPx5 protein is absent, it leaves more luminal H_2_O_2_ that is used by the sperm-nucleus GPx4 (acting here as a disulphide isomerase) to generate disulphide bridges between the sperm nuclear protamines, leading to a greater state of nuclear condensation [69]. The observation, after the 3D-reconstruction, that the *Gpx5^−/−^* sperm show a smoother nuclear surface when compared to the WT sperm which has a “goose-bumps” aspect, is interesting as it distinguishes nuclear domains responding differentially to this oxidation-mediated increased condensation. In particular, the use of different, mild, denaturing treatments (alkaline *versus* reductive denaturation) emphasized the point that even though a mildly oxidized sperm nucleus may appear well-condensed (as for *Gpx5^−/−^* spermatozoa) it is highly susceptible to mild reductive conditions. This is important in clinical practice as clinicians may be misled when using assays such as the aniline blue or the toluidine blue, to determine the level of sperm nuclear condensation as an indicator of sperm nuclear integrity. Therefore, the type of mild denaturation technique chosen will be critical to correctly determine the level of sperm nuclear integrity. These considerations support our credo that a solid evaluation of sperm nuclear integrity/solidity, prior to assisted reproductive technology (ART), should include several additional tests, addressing the issue of DNA fragmentation, DNA oxidation, and nuclear solidity. 

## 4. Materials and Methods

### 4.1. Ethics Statement

Ethics statement: The present study was approved by the Regional Ethics Committee of Animal Experimentation (CEMEA-Auvergne; Authorization CE99-12) and adhered to the current legislation on animal experimentation in France.

### 4.2. Animals

The *Gpx5^−/−^* mice were derived, as described originally, from the C57BL/6 genetic line [41,42]. Mice used in this study (eight mice per genotype) were maintained and housed in temperature-controlled rooms with 12-h light/dark cycles. Mice had ad libitum access to food and water. Nine-month-old mice were culled by cervical dislocation and spermatozoa were collected from the caudal segment of the epididymis.

### 4.3. Immunocytochemistry and Fluorescence in situ Hybridization (FISH) Assays

All immunofluorescence procedures were performed as previously described [35]. Briefly, spermatozoa were resuspended in a decondensing buffer (2 mM DTT and 0.5% triton X-100 in PBS) and incubated for 45 min, at room temperature. After centrifugation at 500× *g* for 5 min, at room temperature, spermatozoa were resuspended in PBS, numbered, and deposited onto a glass plate. For FISH assays, spermatozoa were recovered as described previously [41]. A fraction aliquot of 10 × 10^6^ spz/mL was centrifuged at 560× *g*, for 5 min and re-suspended in 1.25 mL fresh Carnoy’s fixative (3:1 ethanol:acetic acid). This spermatozoa-containing solution was spread on the slides (up to 25,000 spermatozoa/slide) then slides were dried for 1 h, at room temperature (RT), and stored at −20 °C (Superfrost^®^ slides, Thermo Fisher Scientific, Illkirch, France). After 24 h, slides were defrosted at RT and placed in a coplin jar with saline-sodium citrate solution 2X (SSC 2X), for 15 min at 37 °C. Slides were dried for 5 min, at RT, and denatured using NaOH 1.5 N (1 min). Slides were then incubated in a coplin jar with SSC 2X for 30 min, at 70 °C (±1 °C). The coplin jar was left at RT. Slides were successively incubated for 1 min in SSC 0.1X at RT, NaOH 0.07 N at RT, SSC 0.1X at 4 °C, and SSC 2X at 4 °C. Slides were transferred through a series of ethanol washes for 1 min, each starting with 70%, 95%, and finally 100% ethanol. Slides were left to dry at RT. DNA probes were applied to a sterile coverslip, pre-warmed at 37 °C, and sealed using paraffin. Finally, slides were incubated in a dark humidified chamber at 37 °C, for 48 h. Mouse chromosome-painting probes (Metasystems, Altlussheim, Germany) and telomere probes (Panagene, Altlussheim, Korea) were prepared according to the manufacturer’s instructions. After a 48-h incubation period, the slides were washed with SSC 0.4X for 2 min, at 70 °C (±1 °C), and 30 s, in SSC 2X, with Tween 0.05%, and for two successive rinses. Vectashield^®^ with DAPI (Vector Laboratories) was added to each slide to counterstain the sperm cell nucleus. Finally, coverslips were mounted, sealed, and slides were stored in the dark at −20 °C, until observation.

### 4.4. Microscopy

Confocal Z-stacks were captured using a Leica SPE confocal microscope (Leica Microsystems, Wetzlar, Germany) and a 40× oil immersion objective was used for all acquisitions. At least eighty stacks per nucleus were captured and the distance between Z stacks was 0.21 µm. Chromosome territory was assigned after counting not less than three hundred and fifty spermatozoa per chromosome and the percent of spermatozoa presenting a chromosome at one or more given positions was established. A Zeiss microscope Axioplan2 (Carl Zeiss, Oberkochen, Germany) was used to perform these observations.

### 4.5. Image Analysis Measurements of 3D Parameters

All the images were deconvoluted using Huygens software (Scientific Volume Imaging, The Netherlands) before analysis. Spermatozoa volume and surface area were measured using Imaris Version 7.6 software (Bitplane AG, Zurich, Switzerland). The mean of each parameter was calculated with at least 30 spermatozoa.

### 4.6. Statistics

Mann-Whitney and Spearman correlation analyses were performed using GraphPad Prism^®^ software. The difference was considered significant when *p* < 0.001 (**).

## Figures and Tables

**Table 1:** Regional mapping of chromosomes in WT and *Gpx5*^−/−^ mouse sperm nuclei. Chromosome positions are assigned, determined in WT and *Gpx5*^−/−^ mouse sperm nuclei, using FISH. Spermatozoa (n = 350) were counted for each chromosome studied and per genotype. The orange box denote the main position of chromosome.

	WT				*Gpx5−/−*			
	Basal	Apical	Ventral	Dorsal	Basal	Apical	Ventral	Dorsal
Chr 1	27.9	3.4	49.7	19	29.4	8.2	46.3	16.1
Chr 2	25.1	20.2	28.4	26.3	25.5	21	27.5	26
Chr 3	35.9	13.8	31.8	18.5	N.D.			
Chr 7	32.5	9.8	40.3	17.4	29	7.5	47.5	16
Chr 9	29.5	9.8	49	11.7	30	3.8	44.6	21.6
Chr 12	36.8	32.9	13.1	17.2	34.2	27.1	18.9	19.8
Chr 15	21.8	2.8	22.8	52.6	19	7	24	50
Chr 17	57.2	14.1	13.2	15.5	53.8	15.8	16.2	14.2
Chr 18	58.2	22.4	11.2	8.2	57.2	24.3	11.1	7.4
Chr 19	67.2	17	13.6	2.2	61.5	18.4	13.4	6.7
Chr X	7.7	20.7	7.5	64.1	5.3	30.3	4.1	60.3
Chr Y	3.8	29.9	7.2	59.1	4.5	25.4	5.3	64.8

Chr: Chromosome. N.D. not-determined.

**Table 2:** Three-dimensional parameters of sperm nuclei according to treatment and genotype.

	**WT**	***Gpx5*^−/−^**
Average volume (μm^3^)	66	54.8 ^a^
Average Area (μm^2^)	93.9	80.2 ^a^
**Nucleus with NaOH 1.5N Treatment**	**WT**	***Gpx5*^−/−^**
Average volume (μm^3^)	109 ^d^	93.5 ^b,d^
Average Area (μm^2^)	138 ^d^	113.5 ^b,d^
**Nucleus with DTT Treatment (2 mM, 45 min)**	**WT**	***Gpx5*^−/−^**
Average volume (μm^3^)	85.3 ^d^	130.4 ^c,d^
Average Area (μm^2^)	108.4 ^d^	143.3 ^c,d^

Volume and surface area of nuclei were calculated from 3D photographs obtained of Z-stack images, generated with the Imaris software (Bitplane, Switzerland). Nuclei were captured in Z-stacks, using confocal microscopy and subjected to deconvolution (Huygens software, Hilversum, The Netherlands). The resulting distribution of the different parameters are shown in the table for each genotype (WT and *Gpx5*^−/−^). The mean was calculated on thirty spermatozoa per condition. DTT: Dithiothreitol; NaOH: Sodium hydroxide. ‘^a^’ represents *p* < 0.001 for WT no treatment condition; ‘^b^’ represents *p* < 0.001 for WT NaOH condition; ‘^c^’ represents *p* < 0.001 for WT DTT condition; ‘^d^’ represents *p* < 0.001 for no treatment/genotype condition.

## Supplementary Materials

The following are available online at http://www.mdpi.com/2073-4425/9/10/501/s1, Figure S1: Correlation of volume/surface area and size, Table S1: Three-dimensional parameters of sperm chromosomes in WT mouse 3 sperm nucleus.
