# Supplementary material for: Nuclear Integrity but Not Topology of Mouse Sperm Chromosome is Affected by Oxidative DNA Damage
Source: Genes (Basel). 2018 Oct 17;9(10):501. doi: 10.3390/genes9100501 (PMC6210505; doi:10.3390/genes9100501)
Supplement: Supplementary file 1 [file genes-09-00501-s001.pdf]

Mouse sperm chromosome nuclear integrity but not topology is affected by oxidative DNA damage

Champroux A., Damon-Soubeyrand C., Goubely C., Bravard S., Henry-Berger J., Guiton R., Saez F., Drevet JR[&] , Kocer A[&].

GReD "Genetics, Reproduction & Development" Laboratory, UMR CNRS 6293, INSERM U1103, Université Clermont Auvergne, 28 Place Henri Dunant, 63000 Clermont-Ferrand, France

[&]Corresponding authors: ayhan.kocer@uca.fr; joel.drevet@uca.fr

Chromosome with NaOH 1.5N treatment	WT			
n= 30	Size in bp (ENSEMBL)	Position	Average Volume (μm^3)	Average Area (μm^2)
Chromosome 1	197,195,432	Ventral	5.2	20.7
Chromosome 2	182,113,224	Apical	4.12	18.08
		Dorsal	7.2	27.5
		Ventral	8.6	29.2
		Basal	5.9	21.4
Chromosome 3	160,039,680	Basal	5.09	19.77
		Ventral	5.06	19.15
Chromosome 7	145,441,459	Ventral	3.8	15.9
Chromosome 9	124,076,172	Ventral	3.4	14.4
Chromosome 12	120,129,022	Basal	3.45	13.53
		Apical	3.03	13.28
Chromosome 15	104,043,685	Dorsal	2.91	11.77
Chromosome 17	94,987,271	Basal	2.71	11.96
Chromosome 18	90,772,031	Basal	2.75	12.23
Chromosome 19	61,342,430	Basal	2.34	10
Chromosome X	171,031,299	Dorsal	6	20.8
Chromosome Y	91,744,698	Dorsal	2.5	11

Supplemental Table 1

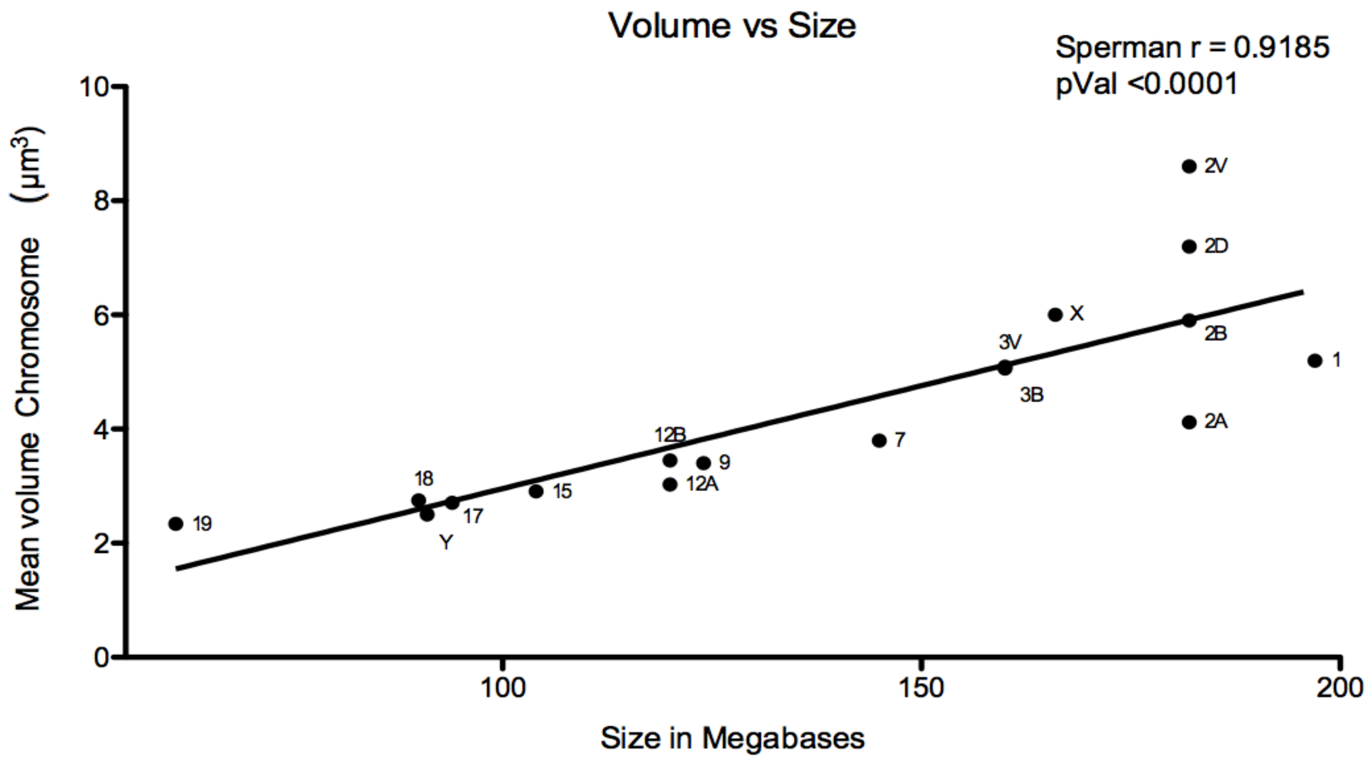

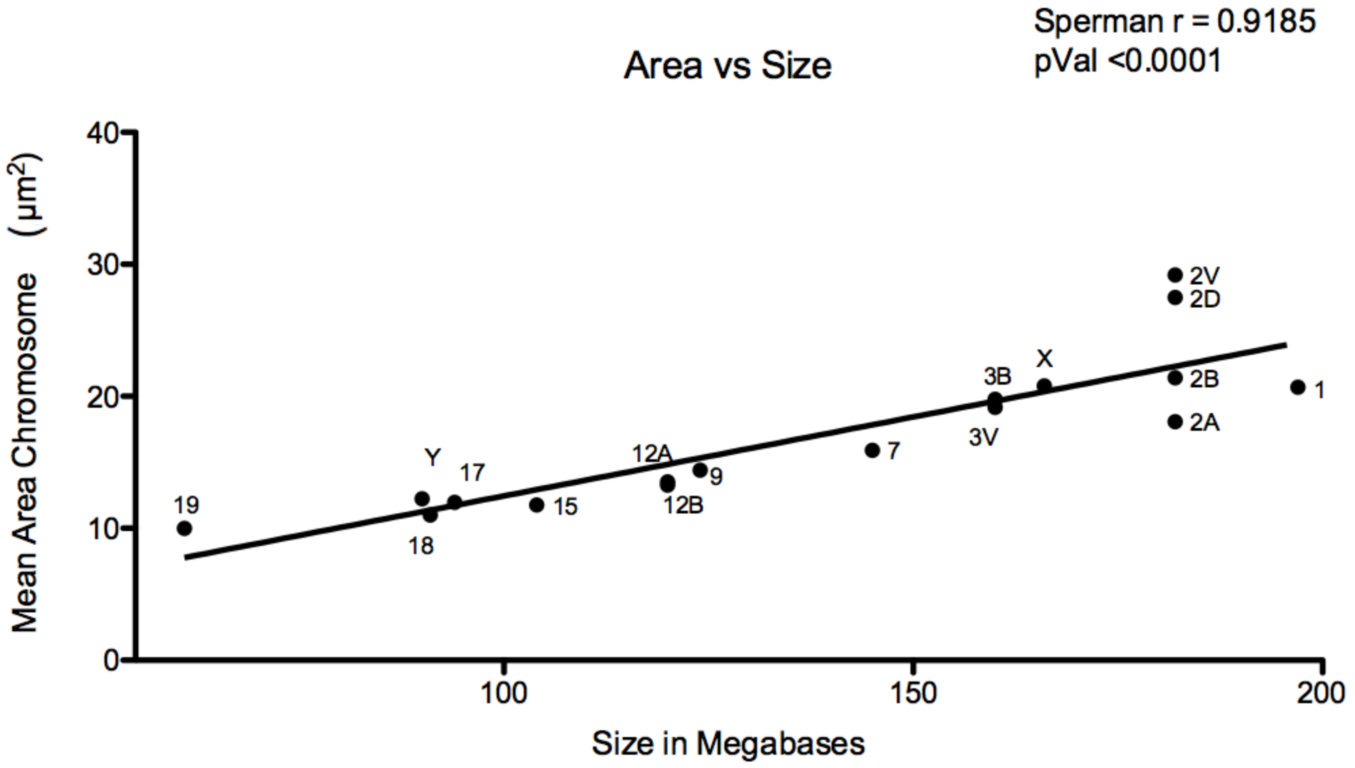

Supplemental Figure 1

Supplemental legends

Supplemental Table 1: Three-dimensional parameters of sperm chromosomes in WT mouse sperm nucleus

Volume and surface area of sperm chromosomes are calculated from 3D photographs obtained of Z-stack images generated with *Imaris* software (Bitplane, Switzerland). Chromosomes are captured in Z-stacks using confocal microscopy and subjected to deconvolution (Huygens software, Netherlands). The resulting distribution of the different parameters are shown in the table. The mean was calculated on 30 spermatozoa per condition. The chromosome size in base pairs (bp) and position are noted. NaOH: sodium hydroxide

Supplemental Figure 1: Correlation of volume/surface area and size

The Spearman correlation graphs showing the linear relationship existing between WT sperm chromosome volume (μm^3) or surface area (μm^2) and chromosome size in base pairs (bp). A: Apical; B: Basal; V: Ventral and D: Dorsal
